# A simulation of energy generation from Jatropha solid residues in a power plant in Jazan city, KSA

**DOI:** 10.1016/j.heliyon.2022.e09352

**Published:** 2022-04-29

**Authors:** Mohamed Hassan

**Affiliations:** Department of Chemical Engineering, College of Engineering, Jazan University, P.O. Box. 706, Jazan 45142, Saudi Arabia

**Keywords:** Solid fuel, Simulation, Power plant, Syngas

## Abstract

A simulation was needed to simulate generating energy from Jatropha plants’ solid wastes in combined cycle power plants. In this study, a simulation was built to simulate energy generation from gasified Jatropha solid wastes. Solid wastes from Jatropha plants' were generated from biodiesel production to produce 1.53 kg/s biodiesel. An assumption was made these solid wastes were gasified to generate energy. Energy production was simulated using DWSIM software. Solid wastes were classified into three categories; cake, shell, and husk. The amount of cake was calculated to be 1.62 kg/s, the shell calculated to be 3.6 kg/s, and the husk was calculated to be 2.16 kg/s. Therefore, the producer gas for each Jatropha solid waste was estimated according to the literature to be 3.65 m^3^/s for the cake, 4.86 m^3^/s for husk, and 8.1 m^3^/s for the shell. The simulation of generating energy from the syngas was validated using experimental data from the literature. Sensitivity analysis was conducted to find the optimum conditions. The results showed that the energy produced from Jatropha waste was higher than that produced from biodiesel. From the simulation, the net energy generated in the gas turbine section was 21.34 MW and from HRSG 12.472 MW. The water flow rate in the compression section of syngas was found to be 10.18 kg/s, which was converted to steam and added more power to the power plant.

## 1 Introduction

Producing oil from Jatropha seeds left a considerable amount of biomass. This biomass is the cake, shell, and husks. The purpose of this paper is to simulate the energy produced from these solid wastes. In this study, an assumption has been made assuming that the solid wastes produced from Jatropha were gasified and introduced to a simulation of a combined cycle power plant to generate energy from a gas turbine and steam turbines. The shell is 35–40 percent of the fruit and the remaining is the seed. The seeds consist of husk and kernels, the husk is around 40% of the seeds, and kernels are around 60% of the seeds. The kernels consist of 50% oil and the remaining is the cake (Singh et al., 2008). Thus, the amount of solids produced from oil extraction is substantial and it can be used as a solid fuel using different techniques such as combustion, gasification, pyrolysis, or anaerobic digestion. (Adinurani et al., 2015) reported that residues from manufactured Jatropha oil can be used for anaerobic digestion. Another study by (Kratzeisen and Müller, 2009), mentioned that the seed shell of Jatropha can be used as fuel with 16–17 MJ/kg calorific value. The properties of residues produced from Jatropha have been studied by (Odetoye et al., 2018). The authors reported that the properties of Jatropha residues are suitable to produce bio-oil. In a similar study by (Kethobile et al., 2020), the authors emphasized that the solid residue of oil production from Jatropha is a great supply of fuel. A study by (Ramírez et al., 2019) showed that the char produced from the pyrolyzed seed cake and seed shell blends can produce energy. In a recent study by (Alherbawi et al., 2021), the authors reviewed most of the studies studied utilizing the cake produced from pressing Jatropha seeds to generate oil for energy. The authors confirmed that the cake can be used to produce bio-oil for energy purposes. Simulating pyrolysis of jatropha residues was studied in many studies, and one of these studies was performed by (Sharma et al., 2016). In this study, the authors studied the pyrolysis of the residues using TGA. (Primandari et al., 2018) reported that solid wastes from Jatropha can be used as briquettes, adsorbent, resin, bioactive compost, and fertilizer.

The gasification process is one of the promising processes to produce gas fuel from solid fuel. A study studying gasification of Jatropha fruit shells was conducted by (Prasad, 2014). In this study, the author studied experimentally the gasification process of fruit shells of Jatropha. Another study by (Agung et al., 2017) studied the simulation of Jatropha shell gasification using Aspen Plus software. The authors predicted the composition of the producer gas produced from the gasification process. The jatropha husk is also one of the biomass products from Jatropha as feedstock for the gasification process. In a recent study by (Pfeil et al., 2020), the husk was characterized to be assessed for use in the gasification process.

A study by (Martinez-hernandez et al., 2014), studied the potential of using biodiesel and solid residues produced from jatropha plants in generating energy. In a recent study by (Piloto-Rodríguez et al., 2020), the authors studied the potential of using the residues produced from Jatropha for energy production. The authors reported that these residues can be gasified or pyrolyzed to produce gas fuel or liquid fuel to generate energy.

Most of the studies in the literature focused on the gasification and pyrolysis processes to generate gas and liquid fuels from solid residues of Jatropha plants. Rare studies regarding producing energy from these fuels. This study focuses on producing energy from the syngas produced from the gasification of the Jatropha solid residues. The solid residues were assumed to be gasified and then introduced to a combined cycle power plant simulation to produce energy.

## 2 Methodology

A case study was discussed utilizing biodiesel in a power plant situated in the south region of KSA. An assumption was made that biodiesel was produced from cultivation Jatropha plants in a region near to the plant. The cultivated area was calculated according to GIS software. The area was estimated to be 228.5 km^2^ and the amount of biodiesel was estimated to be 1.534 kg/s. The biodiesel manufacturing plant was simulated using DWSIM software. Then the biodiesel produced from the simulation was transferred to another simulation for a power plant to blend it with the conventional diesel.

### 2.1 Simulation

The power plant was simulated to predict the amount of energy generated from the blends. In this study, the amount of solid waste produced from the process of pressing the oil from the Jatropha fruits is estimated according to the literature.

The amount of cake according to (Singh et al., 2008) was calculated to be 1.62 kg/s. The amount of the shell was calculated to be 3.6 kg/s and the husk was calculated to be 2.16 kg/s. Therefore, the total of solid residues generated from the production of Jatropha oil is calculated to be 7.38 kg/s. Many technologies can be applied to these wastes to generate liquid or gas fuel using pyrolysis or gasification technologies.

The software used is DWSIM; the thermodynamics model used is Raoult's Law and IAPWS-IF97 steam table. The units used for the simulation consisted of air compressors, mixers, reactors, expanders (turbines), heat exchangers, and pumps. The simple cycle model was validated using real data from a real power plant and the combined cycle model was validated using the literature.

### 2.2 Proximate and ultimate analysis

Proximate and ultimate analysis of Jatropha solid residues were studied in different studies. Table 1 shows a proximate analysis of cake, shell, and husk of the Jatropha residues. While Table 2 shows the ultimate analysis of Jatropha residues.

**Table 1:** Proximate analysis for Jatropha solid residues.

Jatropha solid residues	Proximate analysis				References
	Moisture (%)	Ash (%)	Volatiles (%)	Fixed carbon (%)	
Cake	8.71	4.3	70.92	16.06	(Raja et al., 2010)
	9.22	8.07	74.94	7.84	(Gottipati and Mishra, 2011)
	2.65	3.42	79.8	14.13	(Kim et al., 2013)
	10.03	6.47	72.53	10.97	(Titiloye et al., 2013)
	4.94	4.36	83.96	6.74	(Brunerová et al., 2017)
	3.31	5.99	70.98	19.72	(Jourabchi et al., 2014)
	0.44	1.5	79.2	18.86	(Sharma et al., 2016)
Shell	12.82	6.26	67.38	13.54	(Prasad, 2014)
	8.9	3.8	65	22.3	(Pambudi et al., 2017)
	12.35	14.88	68.73	16.38	(Singh et al., 2008)
	13.4	4.4	82.2		(Hsu et al., 2018)
Shell (dry basis)	-	11.81	66.31	21.88	(Maiti et al., 2014)
Husk	10.75	3.97	71.04	24.99	(Singh et al., 2008)
	8.92	6.58	52.9	31.9	(Murata et al., 2012)
	10.75	3.54	63.4	22.3	(Martinez-hernandez et al., 2014)
	-	3–5	64.9–71	25–31.1	(Navarro-Pineda et al., 2016)
	8.96	3.9	64.9	31.1	(Salaheldeen et al., 2014)
	9.19	4.12	68.71	27.17	(Pfeil et al., 2020)

**Table 2:** Ultimate analysis for Jatropha solid residues.

Jatropha solid residues	Proximate analysis					References
	C (%)	H (%)	O (%)	N (%)	S∗	
Cake	59.17	6.52	33.93	0.38	-	(Raja et al., 2010)
	50.52	6.15	39.41	2.32	-	(Kim et al., 2013)
	44.42	6.23	44.51	4.33	0.51	(Titiloye et al., 2013)
	54.83	7.32	25.3	3.05	0.21	(Brunerová et al., 2017)
	45.75	6.24	38.2	3.56	-	(Jourabchi et al., 2014)
	52.3	6.5	26.8	5.2	-	(Sharma et al., 2016)
	48.80	6.20	33.08	3.85	-	(Prasad, 2014)
Shell	50.9	5.8	39.5	0.8	0.1	(Pambudi et al., 2017)
Shell (dry basis)	42.45	5.12	50.83	1.6	-	(Maiti et al., 2014)
Husk	29.01	4.49	-	1.22	-	(Murata et al., 2012)
	50.3–50.9	5.8–6.6	38.3–39.5	0.2–1.8	0.08	(Navarro-Pineda et al., 2016)
	50.9	5.8	39.5	0.8	0.08	(Salaheldeen et al., 2014)

∗ In some of the literature sulfur was not reported.

### 2.3 Producer gas from gasification

In this study, an assumption was made; that there was a gasification process before adding the fuel to the power plant to generate energy. The producer gas was calculated according to the literature.

(Maiti et al., 2014) studied the gasification process of the Jatropha's shell. The authors confirmed that the gas produced from the downdraft gasifier is used to generate energy and the calorific value of the gas was 5.2 MJm^-3^. The authors reported that the feed of the Jatropha shell was around 15 kg/h. The authors added the range of the flow rate of the producer gas by (m^3^h^−1^) to the biomass consumption rate by (kg h^−1^) was 2.04–2.39 with an average of 2.15. The authors used an equivalence ratio of an average of 0.28, so the gas flow rate is calculated to be 32.25 m^3^h^-1^. The authors added the average CO produced was 17.5%, H_2_ was 9%, CH_4_ was 5.5%, and the remainders were supposed to be N_2_ and CO_2_. In a similar study by (Prasad, 2014), the authors reported that they faced a problem in gasifying the Jatropha shell in a downdraft gasifier. The authors attributed that to the low bulk density of the Jatropha's fruit shell and they added the solution for this problem was to pelletize the Jatroph shell to get higher bulk density. The authors reported the syngas percentages to be H_2_ 6.72%, CO 9.99%, CH_4_ 1.06%, CO_2_ 9.51%, and N_2_ 72.72% with a flow rate of 11.65 g/s. (Pambudi et al., 2017) simulated the gasification of the Jatropha shell. The authors found that the average composition of the producer gas is CO 48.93%, H_2_ 31.49%, CO_2_ 16.86%, and CH_4_ 5.23%.

(Singh et al., 2008) studied the gasification of Jatropha's husks in a downdraft gasifier. The authors discussed two different gas flow rates. The first flow rate obtained was 4.5 m^3^ h^−1^ with 14.08% H_2_, 14.05% CO, 1.86% CH_4_, 57.02% N_2_, and 12.99 (CO_2_+O_2_). The second flow rate obtained was 5.5 m^3^ h^−1^ with 10.62% H_2_, 19.26% CO, 1.71% CH4, 57.08% N_2_, and 11.33 (CO_2_+O_2_).

(Thiagarajan et al., 2018) studied experimentally producing syngas from Jtropha's cake. The authors reported that the average of gas percentages was 5.995% CO, 5.405% H_2_, 1.485% CH_4_, and 9.05% CO_2_, and the remainder by difference is 78.065% N_2_

From the above literature, it can be observed that all Jatropha's residues can be gasified to produce syngas depending on the proximate and ultimate analysis of each residues' component. (Maiti et al., 2014) reported that 32.25 m^3^h^-1^ of syngas can be produced from 5 kg h^−1^ of jatropha's shell. (Prasad, 2014) confirmed the percentages of producer gas including CO_2_ and N_2_. (Singh et al., 2008) reported the amount and percentages of producer gases of Jatropha's husks. (Thiagarajan et al., 2018) reported the percentages of Jatropha's cakes.

In this study, the amount of cake according to (Singh et al., 2008) was calculated to be 1.62 kg/s, for the shell 3.6 kg/s, and husk 2.16 kg/s. Thus, the total of solid residues generated from the production of Jatropha oil is calculated to be 7.38 kg/s. For each 1 kg/h of Jatropha's shell, 2.25 m^3^/h of gas can be produced. With the lack of information about Jatropha's husks and Jatropha cake, an assumption has been made to count the gas as same as the amount of gas produced from the shell. In this study, the amount of cake is 5832 kg/h, the amount of shell calculated to be 12960 kg/h, and the amount of husk calculated to be 7776 kg/h. Therefore, the producer gas for each Jatropha product was estimated to be 1,3122 m^3^/h for the cake, 17,496 m^3^/h for husk, and 29,160 m^3^/h for the shell.

## 3 Results and discussions

The model was validated and then used to simulate the generation of energy from the solid wastes of Jatropha.

### 3.1 Model validation

To validate this model, experimental data was collected from (Niu et al., 2021). Tables 3, 4, 5, and 6 show the data collected from the reference to that used in the simulation. Table 4 shows the composition of the syngas from the reference. The same composition was used in the simulation.

**Table 3:** Gas turbine validation data.

Parameters	(Niu et al., 2021)	This study
Inlet temperature (K)	1505.15	1485.74
Outlet temperature (K)	839.15	857.56
Flue gas flow (kg/s)	70.5	70.5
Combustor efficiency (%)	99	99
Net gas turbine power (MW)	25.06	29.234
Gas turbine efficiency (%)	87	87
Gas turbine outlet pressure (KPa)	101.3	101.3
The pressure ratio of the air compressor	18	18
The efficiency of the air compressor (%)	88	88
The temperature of the air (K)	298.15	298.15

**Table 4:** Gasified gas composition from the model and simulation.

Syngas Components	(Niu et al., 2021)	This study
H_2_%	20.2	20.2
CO%	29.9	29.9
CO_2_%	13	13
CH_4_%	2	2
C_2_H_6_%	0. 9	0. 9
C_2_H_4_%	0.4	0.4

**Table 5:** Syngas compression data from (Niu et al., 2021) and this study.

Syngas parameters	(Niu et al., 2021)	This study
Syngas temperature for the gasification process (K)	1538.15	1538.15
Syngas temperature after the cooling process (K)	393.15	393.15
The first stage compression pressure of kPa	304	304
The second stage compression pressure of kPa	741	741
The third stage compression pressure of kPa	1800	1800
Syngas compressors' efficiencies (%)	80	80
Temperature after first Syngas cooler (K)	399.15	399.15
Temperature after second Syngas cooler (K)	399.15	399.15

**Table 6:** Heat recovery steam generator data.

Syngas Components	(Niu et al., 2021)	This study
The efficiency of high-pressure steam turbine (%)	84	84
The efficiency of low-pressure steam turbine (%)	84	84
Steam high pressure (kPa)	8000	8000
Steam low pressure (kPa)	800	800
Inlet high-pressure steam turbine temperature (K)	753.15	785.46
Inlet low-pressure steam turbine temperature (K)	493.15	500.16
The pressure of the condenser (kPa)	7	7
The efficiency of the pumps (%)	85	85
HPST power plus LPST power (MW)	13.82	12.472
Syngas compressor and water pumps energy (MW)	4.5	5.79

**Figure 1:**
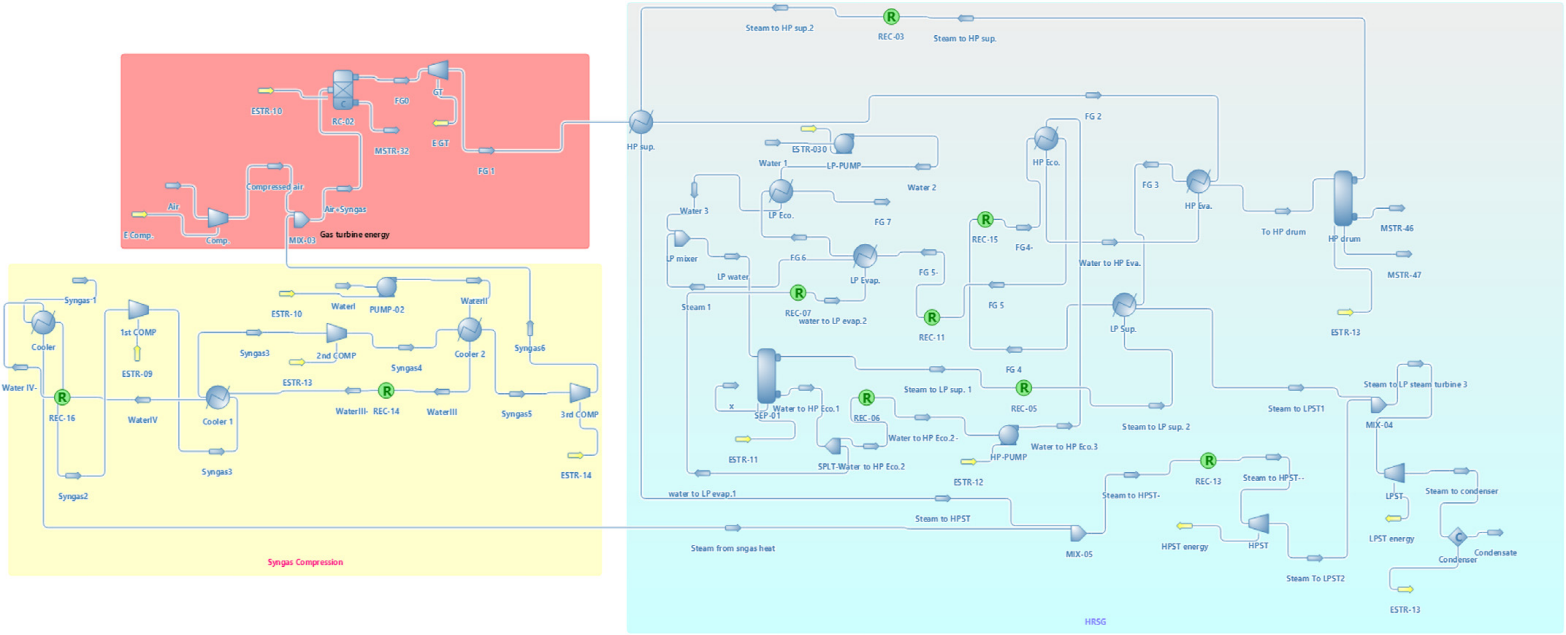
Flowsheet for CCPP.

**Figure 2:**
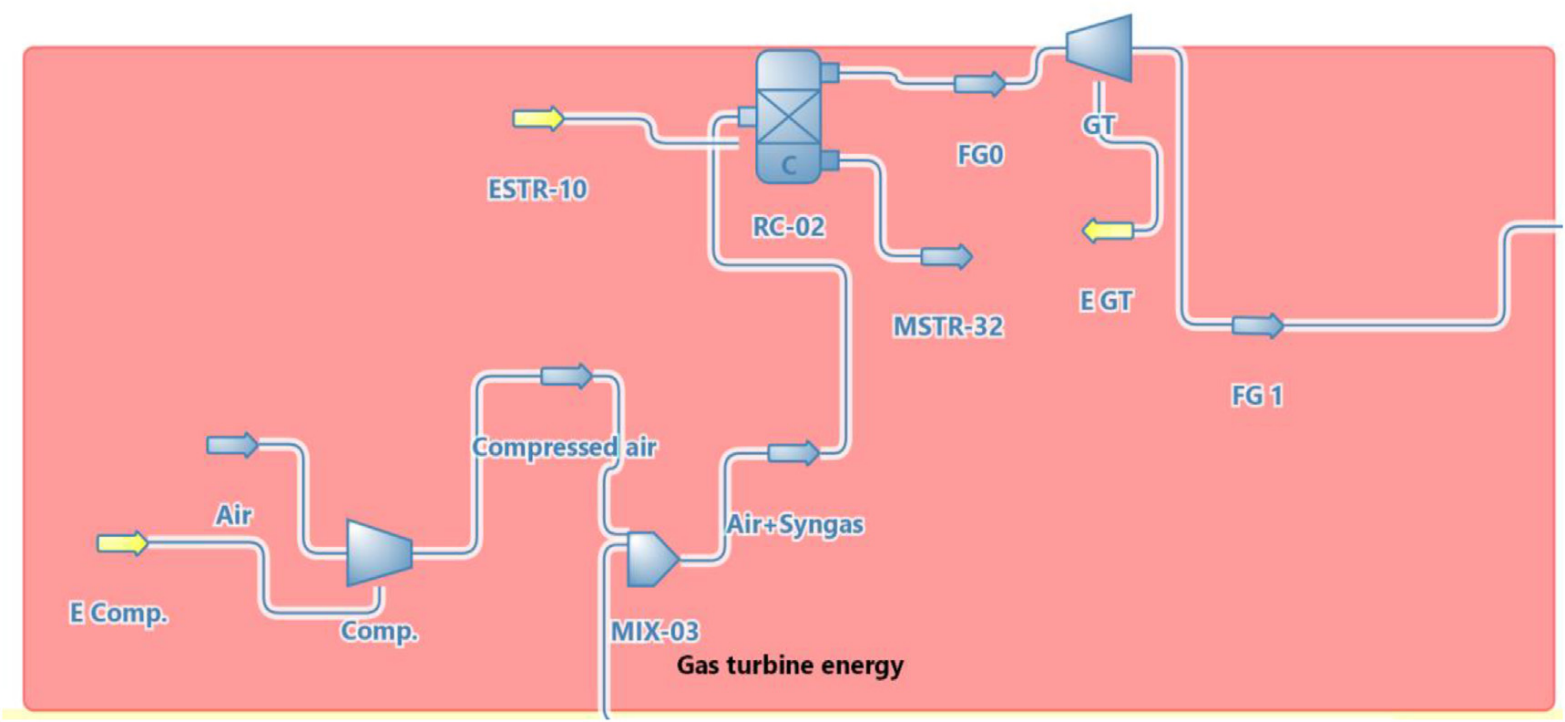
The gas turbine energy section.

**Figure 3:**
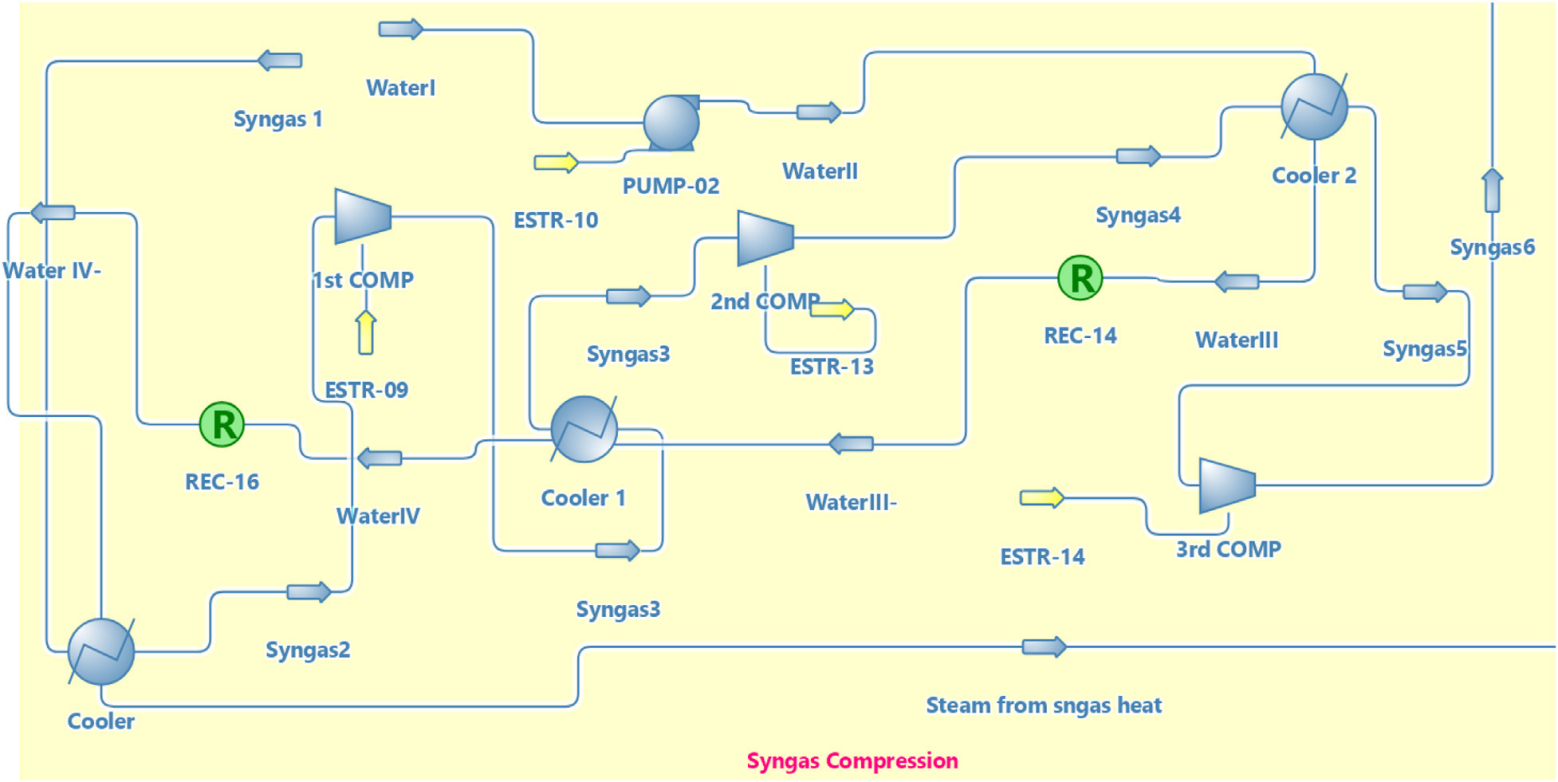
Syngas compression section.

**Figure 4:**
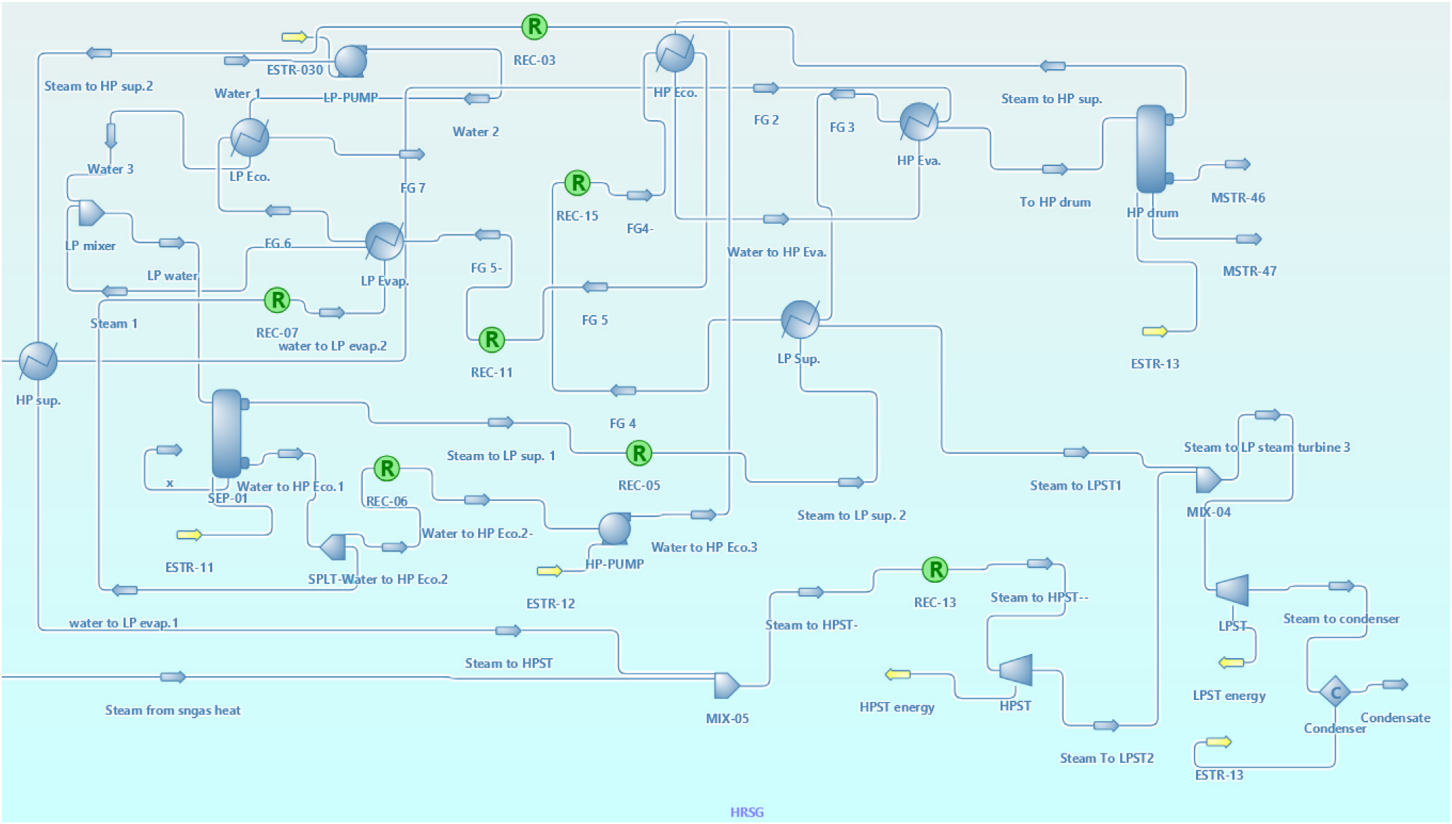
HRSG section.

There are three main sections as shown in Figure 1. The first section is (Gas turbine energy section) as shown in Figure 2 which mainly consists of the air compressor, combustor, and gas turbine. The second section is (Syngas compression section) as shown in Figure 3, which consists of a Syngas cooler, three gas compressors, two intercoolers, and a pump to pump the water. The third section is (the HRSG section) as shown in Figure 4, which consists of two steam turbine loops; one for high pressure and the other for low pressure. Each loop consists of an economizer, evaporator, superheater, steam turbine, pumps, and decanter.

Gas turbine energy section parameters were illustrated in Table 3. The main parameters which were used in the reference (Niu et al., 2021) are; the flue gas flow, combustor efficiency, gas turbine efficiency, gas turbine outlet pressure, the pressure ratio of the air compressor, the efficiency of the air compressor, and air temperature. Then the model used in this study calculated the inlet and outlet temperature of the gas turbine and the net gas turbine power. The model showed very good agreement with the experimental data from (Niu et al., 2021).

The syngas composition which was used in the syngas compression section was illustrated in Table 4. The components of syngas were extracted from (Niu et al., 2021).

In this study, there was an assumption that has been made which is the gas was gasified in a previous process. Table 5 shows the parameters of the syngas from (Niu et al., 2021). The same parameters were used in this study.

Table 6 shows the parameters used for the setting of HRSG. Data extracted from (Niu et al., 2021) and used in this study were the efficiency of high and low-pressure steam turbines, steam's high and low-pressure, condenser's pressure, and pumps' efficiency. The data retrieved from this study are the inlet temperatures of high and low-pressure steam turbines, and the energy consumed by the syngas compressors and pumps. This study showed very good agreement with the data extracted from (Niu et al., 2021).

### 3.2 Sensitivity analysis for validation

**Figure 5:**
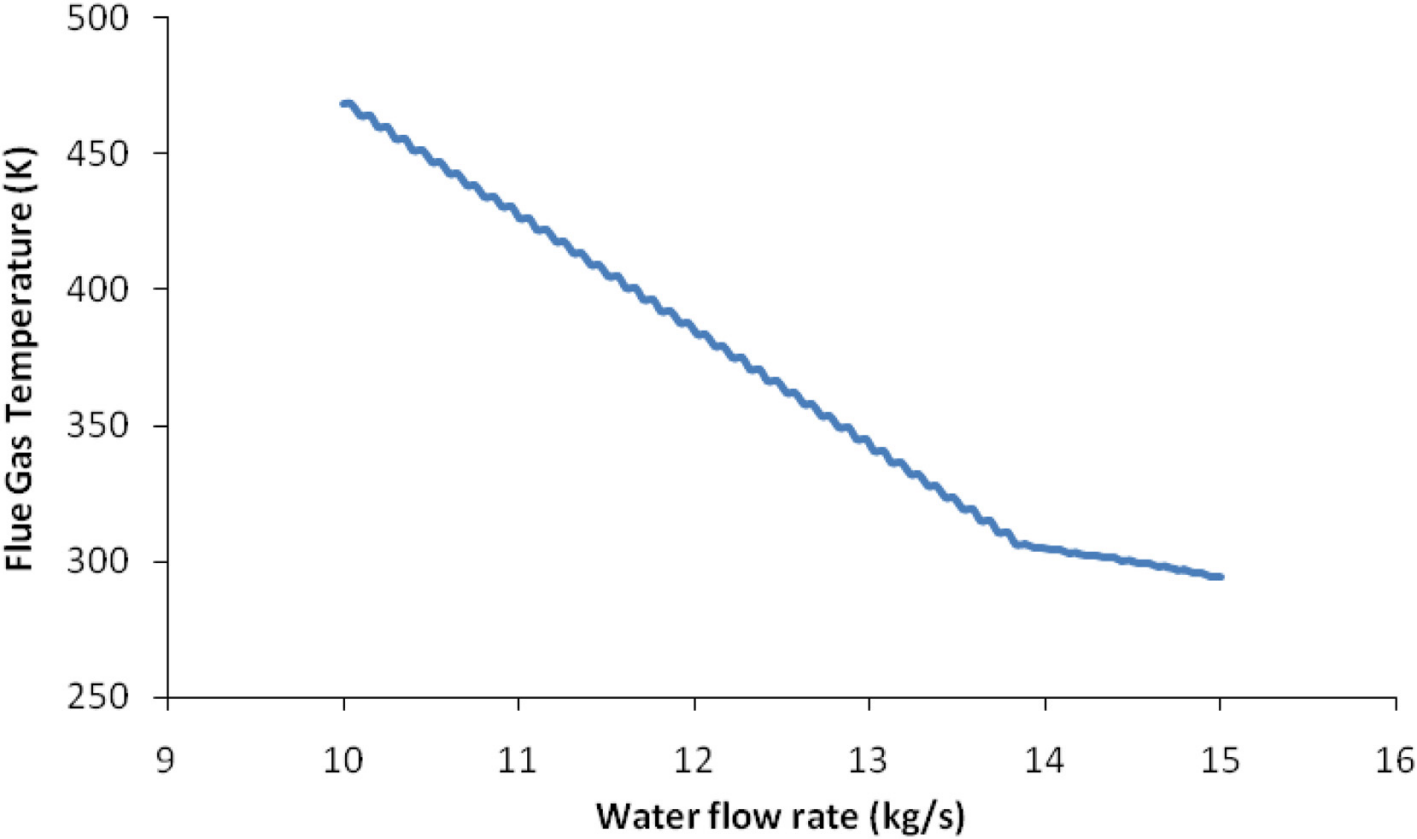
Water flow rate against flue gas outlet temperature.

The water flow rate in the HRSG was calculated according to the temperature outlet of the flue gas to maintain it not decreasing less than 423.15K. Thus, the water was varied by changing the water flow until the flue gas desired temperature was achieved as shown in Figure 5. The best flow rate was calculated by the software to give 10.92 kg/s for 424.1K in the flue gas outlet.

**Figure 6:**
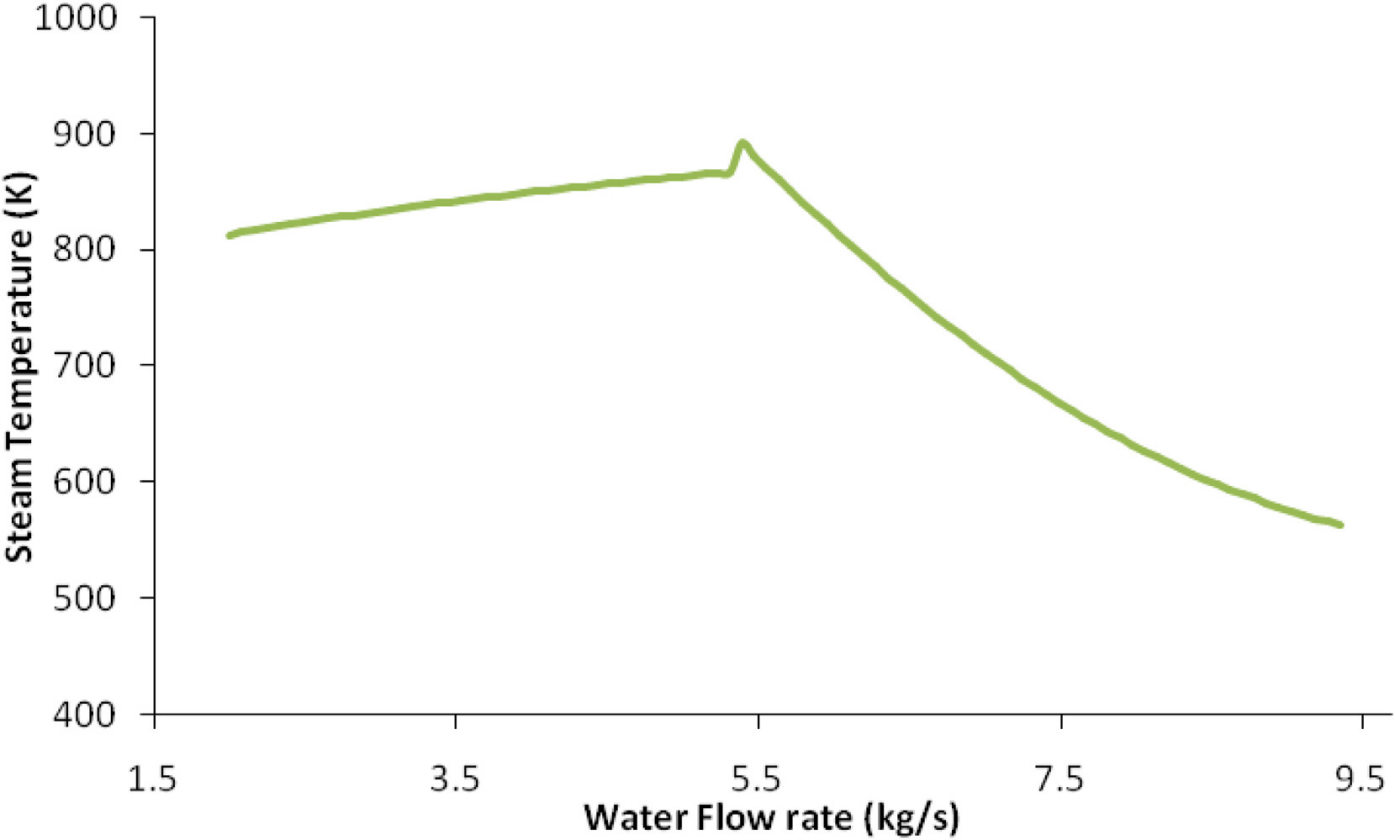
Water flow rate with steam temperature.

**Figure 7:**
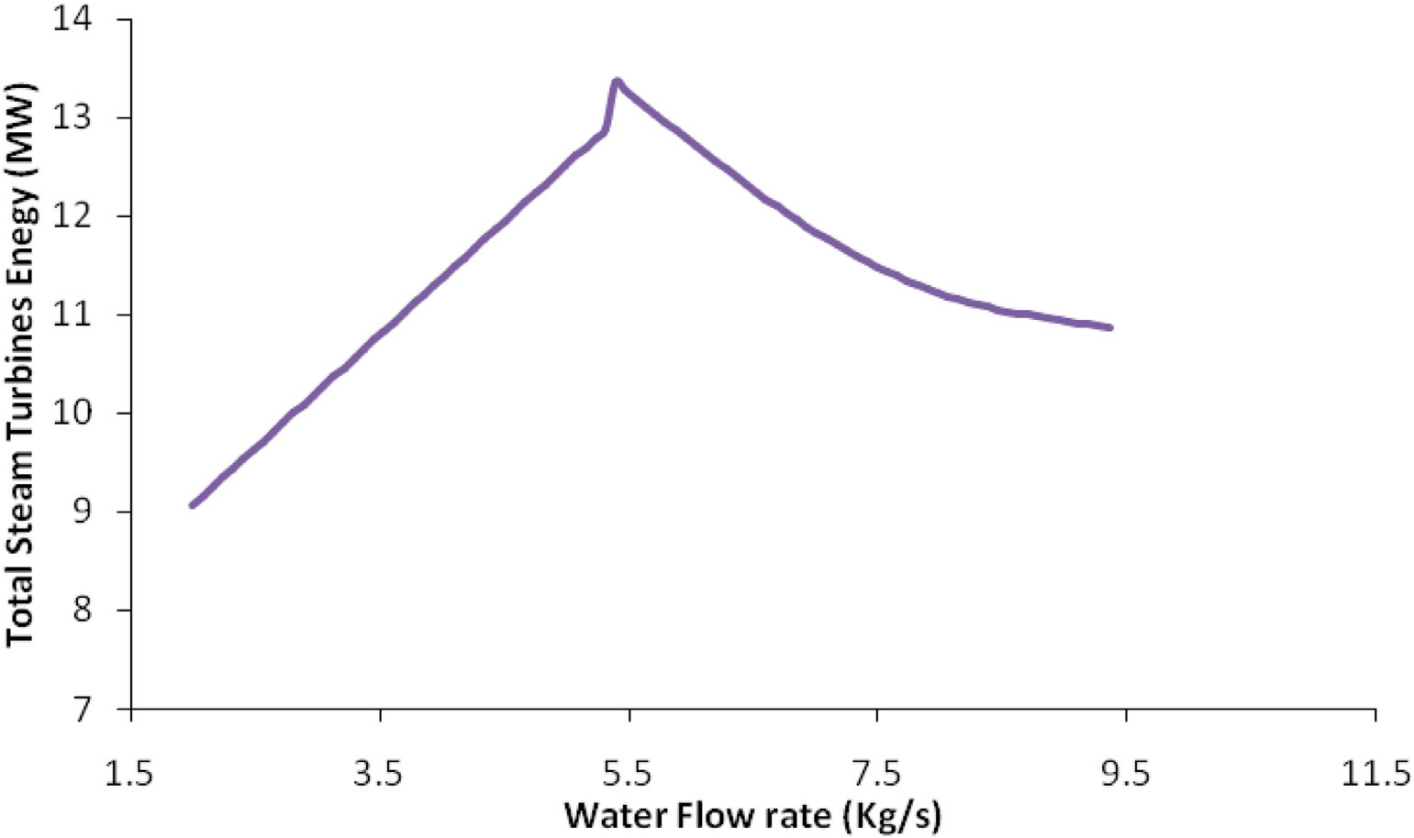
Water flow rate with total steam turbine energy.

Additionally, the water flow rate, which was used to cool the syngas in the syngas compression section, was also varied as shown in Figure 6 to find the best steam turbine energy with the design temperature of the inlet steam turbine around 785.46 K as shown in Figure 7. The water flow rate was found to be 6.28 kg/s.

### 3.3 Sensitivity analysis

**Figure 8:**
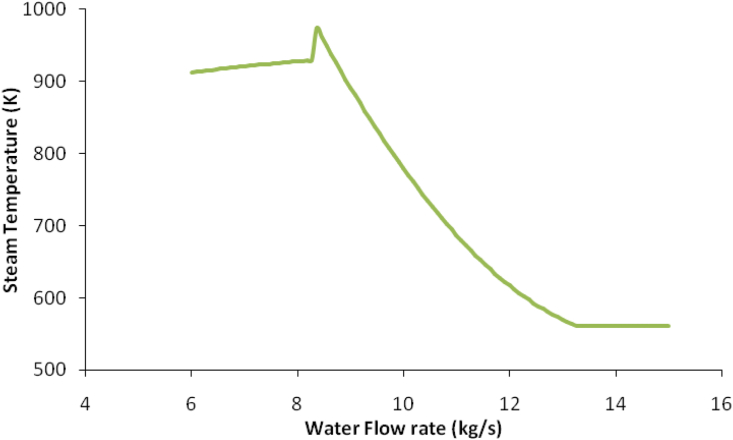
Adding syngas feed.

After validation, Jatropha's syngas was introduced to the process. The syngas generated from Jatropha's husk, cake, and fruit's shell was introduced in three streams and then mixed as shown in Figure 8. The constraints of the simulation are; the flue gas outlet temperature has to be higher than 423.15 K and the temperatures of the inlet and outlet of the high-pressure steam turbine have to be similar to the design temperatures which were used (Niu et al., 2021).

Sensitivity analyses were conducted on water flow rates to find the best water flow to give the best energy with considering the design temperatures for gas turbine inlet temperature and flue gas outlet temperature.

#### 3.3.1 Sensitivity analysis for water flow around the syngas compression section

**Figure 9:**
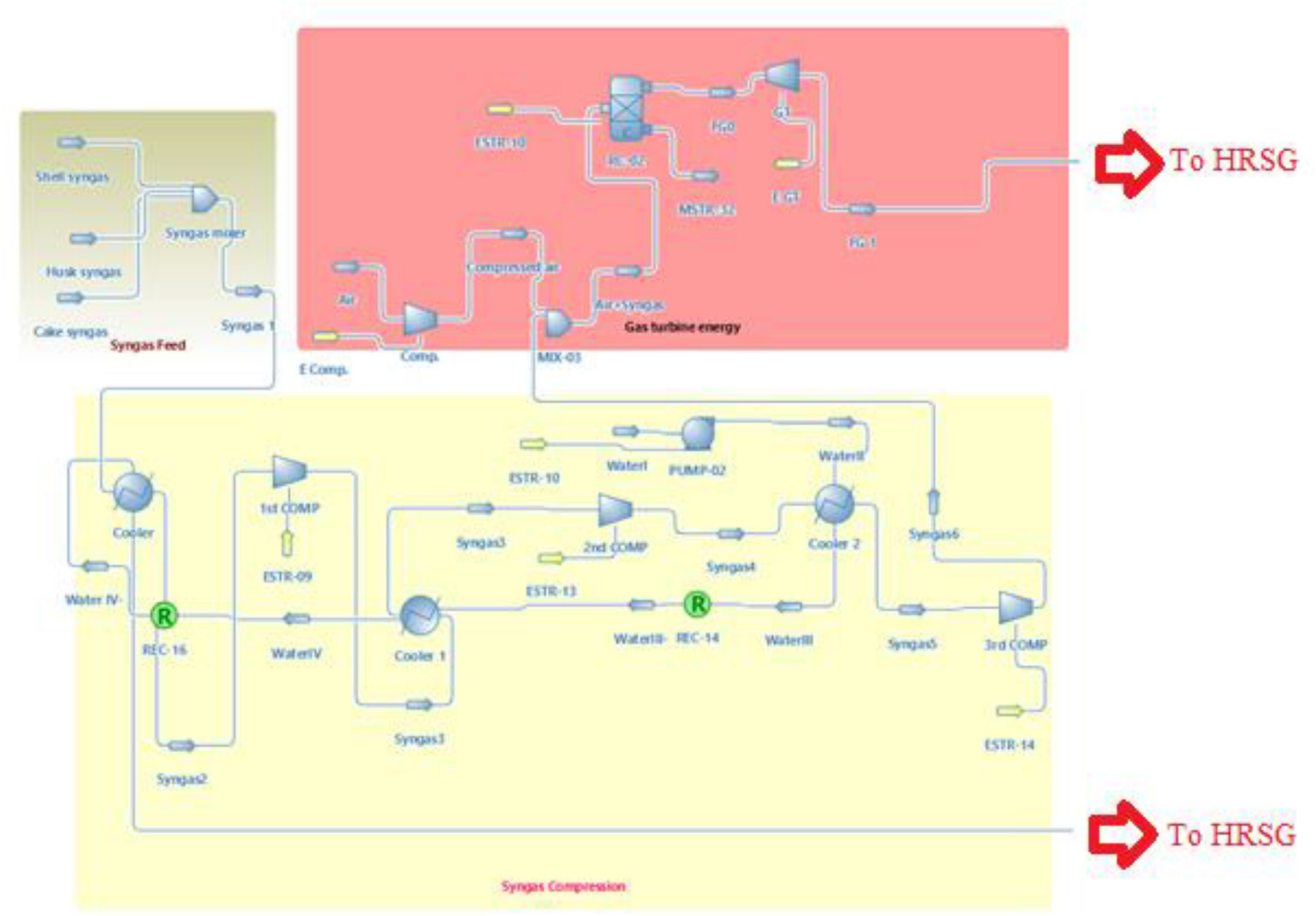
Water flow rate against steam temperature around the syngas compression section.

The water flow rate in the gas compression section was varied to find the high-pressure steam turbine (HPST) inlet temperature around 753.15 K and the inlet temperature of the low-pressure steam turbine (LPST) around 493.15 K. From the sensitivity analysis, the water flow rate was found to be 10.18 kg/s as shown in Figure 9, which gave HPST inlet temperature to be 760.98 K and for LPST inlet temperature to be 484.47 K which they were almost similar to the design temperatures.

#### 3.3.2 Sensitivity analysis for the water flow rate in HRSG

**Figure 10:**
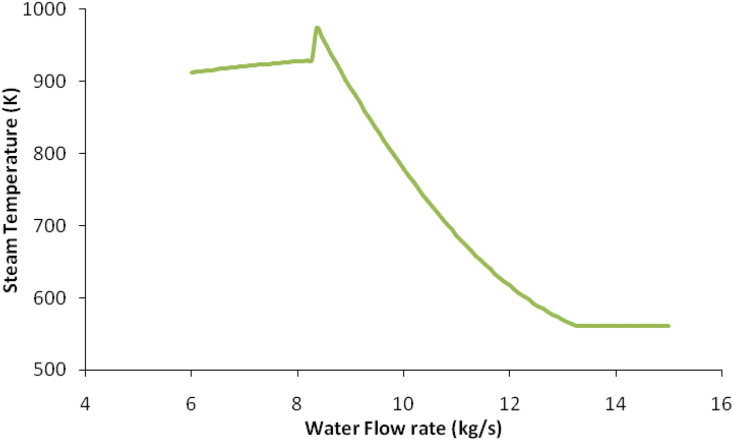
The water flow rate in the HRSG section with flue gas outlet temperature.

The water flow rate in HRSG was varied to calculate the best water flow rate to give the temperature of the flue gas higher than 423.15 K which is the design temperature. The water flow rate was found to be 5.782 kg/s which give the outlet temperature for flue gas 423.199 K as shown in Figure 10.

## 4 Conclusion

Syngas produced from gasified Jatropha solid waste was introduced to a simulation of a combined cycle power plant. The syngas flow was 18.52 Kg/s and consisted of N_2_, CO_2_, H_2_, CH_4_, and CO. The fractions were calculated according to the literature. The temperature of the syngas was assumed to be 1538.15K according to the literature. The syngas was cooled and compressed; the cooling process helped to generate more energy. The syngas was then combusted and introduced to the gas turbine to generate 21.34 MW net powers. Then the flue gas at 998.21 K was introduced to HRSG to create steam to generate power. The power generated in the HRSG was 6.46 MW from the HPST and 5.589 from the LPST. A sensitivity analysis was conducted to vary the water flow rate in the syngas compression section and HRSG section. The water flow rate was found to be 10.18 kg/s in the syngas compression section and 5.782 in the HRSG. The syngas generation was assumed according to the literature. In future work, it is recommended to build a simulation to simulate the syngas production and then to introduce it to the combined cycle power plant.

## Declarations

### Author contribution statement

Mohamed Hassan: Conceived and designed the analysis; Analyzed and interpreted the data; Contributed analysis tools or data; Wrote the paper.

### Funding statement

This research did not receive any specific grant from funding agencies in the public, commercial, or not-for-profit sectors.

### Data availability statement

Data included in article/supplementary material/referenced in article.

### Declaration of interests statement

The authors declare no conflict of interest.

### Additional information

No additional information is available for this paper.
